# Survival of Patients Subjected to Hepatectomy After Spontaneous Rupture of Hepatocellular Carcinoma: A Meta-analysis of High-quality Propensity Score Matching Studies

**DOI:** 10.3389/fonc.2022.877091

**Published:** 2022-05-19

**Authors:** Xiaozhun Huang, Chenyang Jia, Lin Xu, Xinyu Bi, Fengyong Lai, Zhangkan Huang, Xiaoqing Li, Xin Yin, Yong Ni, Xu Che

**Affiliations:** ^1^ Department of Hepatobiliary Surgery, National Cancer Center/National Clinical Research Center for Cancer/Cancer Hospital & Shenzhen Hospital, Chinese Academy of Medical Sciences and Peking Union Medical College, Shenzhen, China; ^2^ Department of Hepatopancreatobiliary Surgery, Second People’s Hospital, First Affiliated Hospital of Shenzhen University, Shenzhen, China; ^3^ Department of Hepatobiliary Surgery, National Cancer Center/National Clinical Research Center for Cancer/Cancer Hospital, Chinese Academy of Medical Sciences and Peking Union Medical College, Beijing, China; ^4^ Department of Intervention, National Cancer Center/National Clinical Research Center for Cancer/Cancer Hospital & Shenzhen Hospital, Chinese Academy of Medical Sciences and Peking Union Medical College, Shenzhen, China

**Keywords:** hepatocellular carcinoma, hepatectomy, meta-analysis, rupture spontaneous, survival, propensity score matching, survival of rHCC vs. nrHCC

## Abstract

**Background:**

The spontaneous rupture of hepatocellular carcinoma (HCC) is associated with high mortality rates, and liver resection can provide better outcomes than other available treatments. However, the survival length of patients subjected to hepatectomy after spontaneous rupture of hepatocellular carcinoma remains controversial.

**Method:**

Articles reporting the comparison of the survival outcome between patients with rupture HCC (rHCC) and non-rupture HCC (nrHCC) from the inception until December 31, 2021 by PubMed, Web of Science, OVID, and the Cochrane Library databases were included. The high-quality propensity score matching analysis was used to investigate the impact of rupture on disease-free survival (DFS) and overall survival (OS) between the rHCC and nrHCC group with no heterogeneity.

**Result:**

A total of 606 patients from six cohort studies were included. The major baseline characteristics of the eligible patients were well balanced between rHCC and nrHCC group. The 1-, 3-, and 5-year hazard ratios of DFS were 3.45 (95% confidence interval [CI] 2.54–4.68), 3.63 (95% CI 2.87–4.60), and 3.72 (95% CI 2.93–4.72), respectively. The 1-, 3-, and 5-year hazard ratios of OS were 5.01 (95% CI 3.26–7.69), 5.49 (95% CI 4.08–7.39), and 4.20 (95% CI 3.20–5.51), respectively.

**Conclusion:**

The present meta-analysis demonstrated that the DSF and OS were significantly shorter in the rHCC group than in the nrHCC group, thus revealing that spontaneous HCC rupture was a predictor of poor survival.

## Introduction

Spontaneous rupture of hepatocellular carcinoma (HCC) is associated with the highest mortality rates in HCC patients because of uncontrollable bleeding associated with hemorrhagic shock, altered liver function due to cirrhosis, challenging diagnosis, and delayed management (1). Spontaneous rupture occurs in 3%–26% of all HCC patients, with a reported high mortality rate ranging from 32% to 67% (2–4).

Evidence of long-term surgical outcomes of patients with spontaneous ruptured HCC (rHCC) treated with hepatic resection is reported when resection is carefully performed in certain patients. However, these studies provide limited results because it is still unclear and inconsistent whether rHCC impacts tumor recurrence and the length of patient survival (5–7). Although some studies revealed that surgical procedures provide better outcomes than other treatments in patients with rHCC (2, 8, 9), others reported that the prognosis is worse in patients with spontaneous rHCC than in those with non-ruptured HCC (nrHCC), even after R0 hepatic resection (10, 11). However, some studies found that the length of overall survival (OS) and/or disease-free survival (DFS) was similar in patients with rHCC and those with nrHCC (4, 6, 12). A recent meta-analysis considering observational studies showed that the spontaneous tumor rupture is a prognostic risk factor for HCC after hepatic resection (13). However, the pooling of low-quality studies can compromise the strength of the results (14). Importantly, these studies considered small cohorts, and the outcomes remain controversial, because the baseline of the included sample was heterogeneous and unbalanced.

Randomized clinical trials (RCTs) on patients with rHCC are difficult to perform because of the suddenness, hemodynamic instability and active bleeding after tumor rupture. However, evidence is available showing estimates derived from high-quality nonrandomized comparative studies such as propensity score matching (PSM) studies, which may be similar to those derived from RCTs (15). Therefore, this work represents a meta-analysis that reviews the currently high-quality available PSM data comparing rHCC with nrHCC to evaluate the impact of tumor rupture on the length of patient survival after hepatectomy.

## Materials and Methods

The present meta-analysis was performed according to the criteria defined by the Preferred Reporting Items for Systematic Reviews and Meta-analyses statement (16).

### Data Source and Search Strategy

A literature search of the PubMed, Web of Science, OVID, and the Cochrane Library databases was performed to find relevant articles, with no restriction of region. The search included literature published until January 14, 2020 with no lower date limit. A second search was performed in case of new published relevant articles on December 31, 2021. Medical subject headings combined with free text words were used to search RCT and observational studies. The following medical heading terms and their combinations in titles and abstracts were considered: hepatocellular carcinoma AND spontaneous OR rupture. All the collected articles were evaluated. The “related articles” function was also used to broaden the search. In addition, the reference lists of all the collected articles were manually screened to identify additional studies. The most recent or complete report was considered in cases of multiple reports describing the same patient population. The literature search was independently performed by two researchers; any disagreement was resolved by the adjudicating senior authors.

### Inclusion and Exclusion Criteria

The inclusion criteria were the following: (a) all published articles reporting the comparison of postoperative and survival outcomes between rHCC and nrHCC. Only studies designed with PSM were further evaluated; (b) pathological confirmation of HCC. The exclusion criteria were the following: (a) lack of reporting relevant outcomes based on available data or inability to calculate them; (b) non-human experimental study design; (c) types of study other than RCTs and observational studies including editorials, letters to the editor, review articles, and case reports.

### Data Extraction and Study Outcomes

Titles and abstracts of the collected articles were evaluated after the removal of duplicates and a sequential exclusion was performed according to the eligibility criteria. The complete text was examined independently by two investigators in case of uncertainties after the screening of the titles and abstracts, and discrepancies, if any, were resolved by consensus. The primary outcomes were 1-, 3-, and 5-year OS and DFS.

### Quality Assessment and Statistical Analysis

The bias in publication was evaluated using the Stata version12.0 software (Stata Corporation, College Station, TX, USA). The available data were centrally checked for completeness, plausibility, and integrity before the compilation into a single database. The methodological quality of the retrospective studies was assessed using the modified Newcastle–Ottawa scale (17, 18), which includes three factors: patient selection, comparability of the study groups, and outcome assessment. A score of 0–9 was assigned as stars to each study. Observational studies with six or more stars were considered of high quality. Discrepancies, if any, were resolved by consensus. The meta-analysis was performed using the Review Manager 5.3 software (Cochrane Collaboration, Oxford, UK). Continuous and dichotomous variables were respectively expressed as weighted mean difference (WMD) and odds ratio (OR). Results were reported with 95% confidence intervals (CI). Statistical heterogeneity among the included studies was assessed using the chi-squared test with the significance set at a value of *P* < 0.05, and heterogeneity was quantified using the *I* ^2^ statistic. The random effects model was used for pooled analyses in case of significant heterogeneity among the included studies, otherwise, the fixed effects model was used (19). Bias in publication was tested using the Stata version12.0 software (Stata Corporation, College Station, TX, USA).

## Results

### Search Results

**Figure 1:**
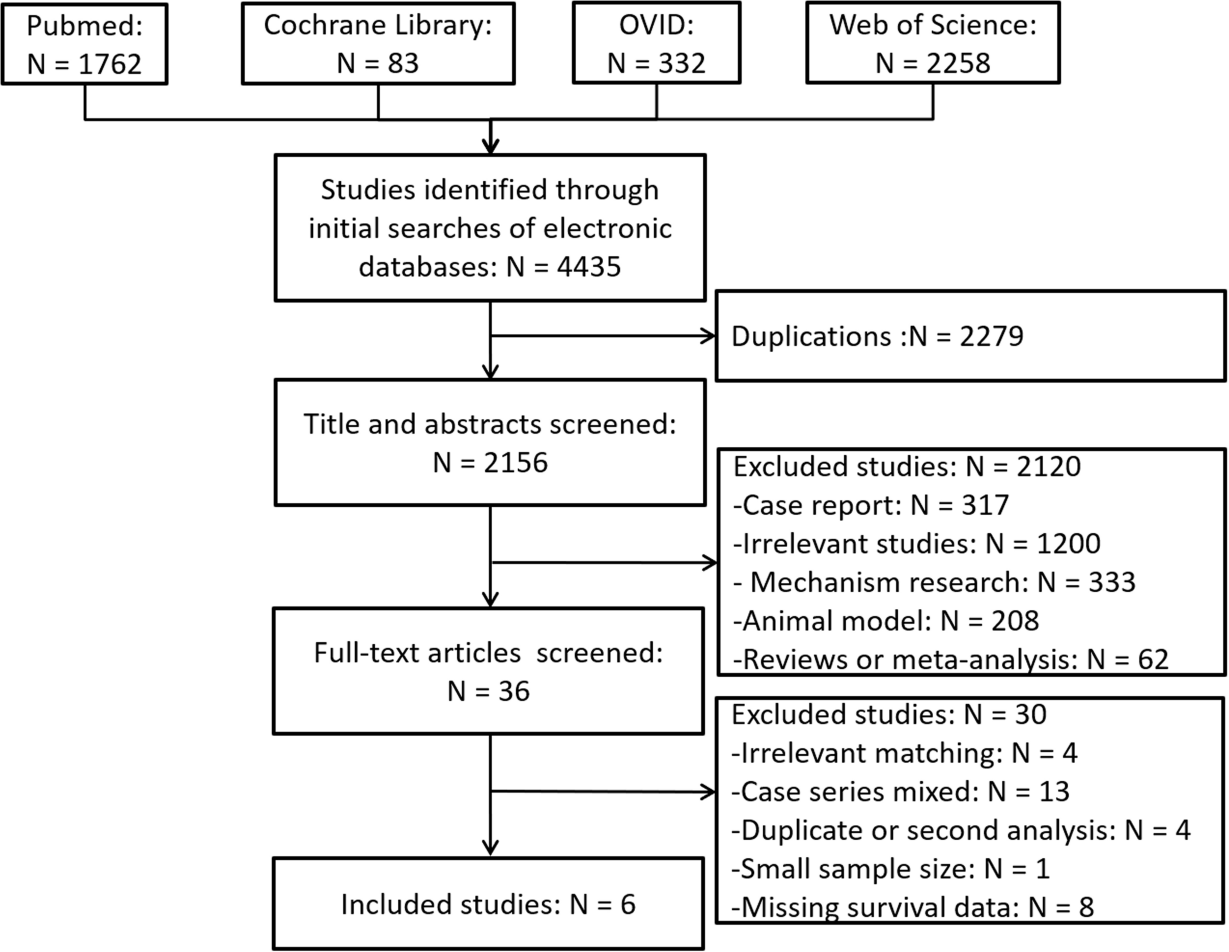
Schematic illustration of the literature search and study-selection criteria.

The schematic illustration of the literature search and the criteria of study selection is shown in **Figure 1**. A total of 4435 articles were collected after an initial search in the biomedical databases. Among these, 2279 were removed because of duplication, and 2156 articles were excluded because irrelevant for the present study after reviewing the titles and/or abstracts. The full text of 36 articles was evaluated, leading to the exclusion of the following articles: two studies available as abstracts only, 13 case reports with inappropriate control groups, 4 studies with duplicate or secondary analyses, 8 studies lacking research data, 1 study with a small rHCC group and 4 studies with a nonmatching design. The remaining 6 studies (7, 20–24) that compared rHCC with nrHCC after hepatectomy using PSM were included in the meta-analysis. No additional studies were identified by the manual screening of the reference lists of these 6 studies and review articles. The agreement between the two reviewers was 98.4% for both the study selection and the quality assessment of the trials.

### Characteristics of the Included Studies

The characteristics of the 6 studies included in the meta-analysis are listed in **Table 1**. All studies were published between 2014 and 2020. The sample size in each individual study ranged from 28 to 178, with a total of 606 patients, and the overall cohort included 269 (44.4%) and 337 (55.6%) patients with rHCC and nrHCC, respectively. The major baseline characteristics of the eligible patients were well balanced between the two groups, with no significant differences in patient demographics (**Table 2**). The meta-analysis using the fixed effects model found no significant difference between the rHCC and nrHCC group regarding the gender ratio (OR, 1.27, 95% CI: 0.80–2.04, *P* = 0.31, *I* ^2^ = 0, **Figure S1A**), age (WMD, -0.62, 95% CI: -1.96–0.72, *P* = 0.37, *I* ^2^ = 0, **Figure S1B**), rate of liver cirrhosis (OR, 0.98, 95% CI: 0.64–1.50, *P* = 0.92, *I* ^2^ = 0, **Figure S1C**) and rate of Child-Pugh classification A (OR, 0.71, 95% CI: 0.39–1.31, *P* = 0.27, *I* ^2^ = 0, **Figure S1D**). In addition, no significant difference between the nHCC and nrHCC groups was found regarding the rate of macrovascular invasion (OR, 1.10, 95% CI: 0.73–1.66, *P* = 0.65, *I* ^2^ = 0, **Figure S2A**), microvascular invasion (OR, 1.22, 95% CI: 0.84–1.79, *P* = 0.30, *I* ^2^ = 0, **Figure S2B**), rate of solitary nodule (OR, -0.04, 95% CI: -0.12–0.04, *P* = 0.31, *I* ^2^ = 30, **Figure S2C**) and tumor size (WMD, 0.22, 95% CI: -0.43–0.86, *P* = 0.51, *I* ^2^ = 4, **Figure S2D**).

**Table 1:** Summary of characteristics of included studies.

Study	Location/year	Number of patients		Age		Sex Ratio(Male : Female)		Childs-Pugh(A:B)		Tumor size (cm)		Macrovascular invasion		Microvascular invasion		Tumor number(solitary:multiple)	
		rHCC	nrHCC	rHCC	nrHCC	rHCC	nrHCC	rHCC	nrHCC	rHCC	nrHCC	rHCC	nrHCC	rHCC	nrHCC	rHCC	nrHCC
Sada H	Japan/2016	14	14	60.9 ± 3.7	62.0 ± 3.7	13:1	13:1	9:5	11:3	9.6 ± 1.7	10.4 ± 1.7	8	12	NR	NR	8:6	9:5
Zhu Q	China/2019	89	89	50.1 ± 7.83	50 ± 7.46	81:8	77:12	83:6	85:4	8.1 ± 4.18	7.9 ± 6.37	50	43	65	60	52:37	59:30
Lee HS	Korea/2014	18	37	53 (40-78)	52 (32-71)	15:3	28:9	NR	NR	6.1 (3.0-15.0)	6.5 (0.9-18.0)	8	13	12	26	12:6	17:20
Chua DW	Singapore/2019	49	98	64 (56-72)	66.3 (58-73)	42:7	86:12	37:12	77:21	8.5 (5.5-11.0)	7.65 (4.0-12.0)	7	11	27	47	NR	NR
Tanaka S	Japan/2016	42	42	65 (48-79)	65 (47-79)	36:6	37:5	40:2	41:1	5.4 (2.2-18.0)	4.4 (2.0-18.0)	2	3	NR	NR	35:7	35:7
Ruan S	China/2020	57	57	50.7 ± 13.9	53.3 ± 13.0	48:9	43:14	57:0	57:0	8.1 ± 4.4	7.1 ± 4.4	52	54	23	21	50:7	56:1

rHCC, ruptured hepatocellular carcinoma; nrHCC, non-ruptured hepatocellular carcinoma; NR, not reported.

**Table 2:** Meta-analysis results of all available studies in population characteristics of the included studies.

Baseline	No. Cohorts	No. Patients		Heterogeneity test		Model	OR/WMD	95%CI	*P*
		rHCC	nrHCC	*I* ^2^	*P*				
Sex ratio	6	269	337	0	0.86	Fixed	1.27	0.80-2.04	0.31
Age	6	269	337	0	0.81	Fixed	-0.62	-1.96-0.72	0.37
Liver cirrhosis	5	212	280	0	0.79	Fixed	0.98	0.64-1.50	0.92
Rate of Child-Pugh A	4	194	243	0	0.93	Fixed	0.71	0.39-1.31	0.27
Macrovascular invasion	6	269	337	0	0.43	Fixed	1.10	0.73-1.66	0.65
Microvascular invasion	4	213	281	0	0.92	Fixed	1.22	0.84-1.79	0.30
Solitary nodule	5	220	239	30	0.22	Fixed	-0.04	-0.12-0.04	0.31
Tumor size	6	269	337	4	0.39	Fixed	0.22	-0.43-0.86	0.51

rHCC, ruptured hepatocellular carcinoma; nrHCC, non-ruptured hepatocellular carcinoma; OR, odds ratio; WMD, weighted mean difference; CI, confidence intervals.

### The Methodological Quality of the Included Studies

The potential sources of bias among the studies were evaluated using the modified Newcastle–Ottawa scale. The quality was generally high in all the included studies (**Table 3**), with scores of 9/9 in 3 studies (7, 22, 24), while 3 studies (20, 21, 23) achieved a score of 8/9. The data related to the follow-up duration were available in 3 studies (7, 22, 24), while other three studies (20, 21, 23) report only data on the follow up modality.

**Table 3:** Risk of bias using the modified Newcastle-Ottawa Scale.

Study	Selection				Comparability	Outcome			Score
	Representativeness of exposed cohort	Selection of non-exposed cohort	Exposure	Outcome of interest not present at start	Comparability of nHCC vs nrHCC	Assessment of outcome	Follow-up	Adequacy of follow-up	
Sada H	Truly representative	Same	Surgical records	Yes	Restricted, matched	Record linkage	Yes	Unclear	8★
Zhu Q	Truly representative	Same	Surgical records	Yes	Restricted, matched	Record linkage	Yes	Unclear	8★
Lee HS	Truly representative	Same	Surgical records	Yes	Restricted, matched	Record linkage	Yes	Complete	9★
Chua DW	Truly representative	Same	Surgical records	Yes	Restricted, matched	Record linkage	Yes	Complete	9★
Tanaka S	Truly representative	Same	Surgical records	Yes	Restricted, matched	Record linkage	Yes	Complete	9★
Ruan S	Truly representative	Same	Surgical records	Yes	No restrictions, not matched	Record linkage	Yes	Complete	8★

rHCC, ruptured hepatocellular carcinoma; nrHCC, non-ruptured hepatocellular carcinoma. ★: scores.

### Survival Analysis

**Figure 2:**
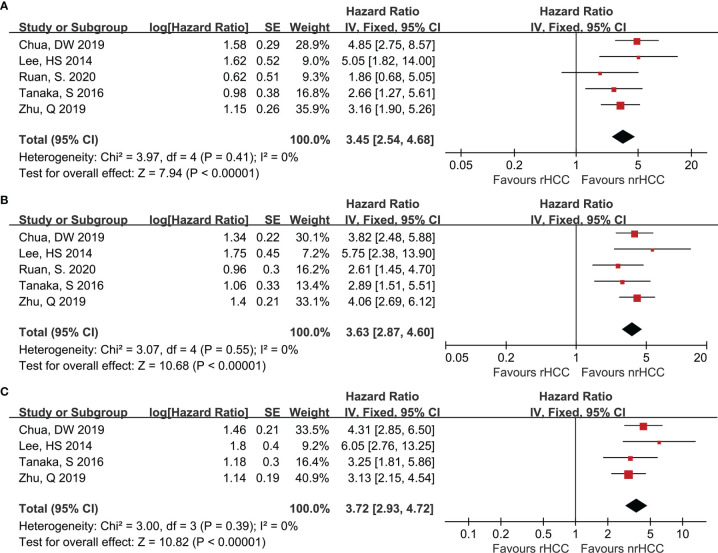
Forest plots for DFS. **(A)** Forest plot for 1-year DFS indicates significantly decreased DFS in the rHCC group as compared with that in the nrHCC group (HR, 3.45; 95% CI: 2.54–4.68). **(B)** Forest plot for 3-year DFS indicates significantly decreased DFS in the rHCC group (HR, 3.63; 95% CI: 2.87–4.60). **(C)** Forest plot for 5-year DFS indicates significantly decreased DFS in the rHCC group (HR, 3.72; 95% CI: 2.93–4.72).

Data on 1- and 3-year DFS were reported in 5 studies (7, 20, 22–24) for the rHCC and nrHCC group using hazard ratios (HR), while 5-year DFS was reported in 4 studies (7, 20, 22, 23). The DFS was lower in the rHCC group than in the nrHCC group; the HR were 3.45 (95% CI 2.54–4.68), 3.63 (95% CI 2.87–4.60), and 3.72 (95% CI 2.93–4.72) for the 1-, 3-, and 5-year DFS, respectively (**Figures 2A-C**). No heterogeneity was found among the 1-, 3-, and 5-year DFS (*I* ^2^ = 0, *P *< 0.00001; *I* ^2^ = 0, *P* < 0.00001; and *I* ^2^ = 0, *P* < 0.00001, respectively).

**Figure 3:**
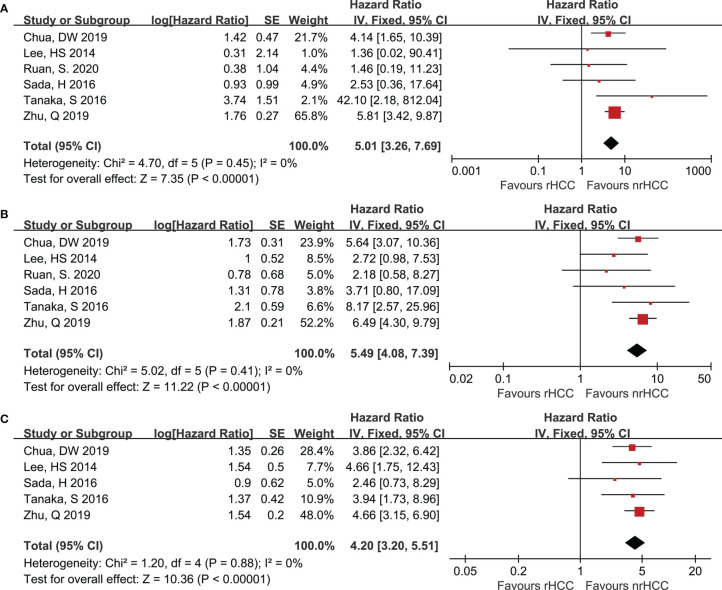
Forest plots for OS. **(A)** Forest plot for 1-year OS indicates significantly decreased OS in the rHCC group as compared with that in the nrHCC group (HR, 5.01; 95% CI: 3.26–7.69). **(B)** Forest plot for 3-year OS indicates significantly decreased OS in the rHCC group (HR, 5.49; 95% CI: 4.08–7.39). **(C)** Forest plot for 5-year OS indicates significantly decreased OS in the rHCC group (HR, 4.20; 95% CI: 3.20–5.51).

All the included trials reported the OS data when the data from the 6 studies were pooled. Six studies (7, 20–24) reported the 1- and 3-year OS for the rHCC and nrHCC group, while 5 studies (7, 20–23) reported the 5-year OS. The meta-analysis revealed that the 1-, 3-, and 5-year OS rates were significantly lower in the rHCC group than in the nrHCC group (**Figures 3A-C**); the HR were 5.01 (95% CI 3.26–7.69), 5.49 (95% CI 4.08–7.39), and 4.20 (95% CI 3.20–5.51) for the 1-, 3-, and 5-year OS, respectively. No heterogeneity was found in the data related to the 1-, 3-, and 5-year OS (*I* ^2^ = 0, *P *< 0.00001; *I* ^2^ = 0, *P* < 0.00001; and *I* ^2^ = 0, *P* < 0.00001, respectively).

### Sensitivity Analysis and Publication Bias

**Figure 4:**
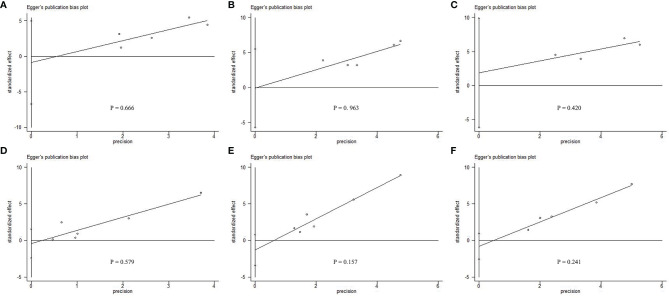
Egger linear regression test does not indicate any evidence of publication bias. Egger linear regression with pseudo 95% confidence limits using **(A)** 1-year DFS, **(B)** 3-year DFS, **(C)** 5-year DFS, **(D)** 1-year OS, **(E)** 3-year OS, and **(F)** 5-year OS, revealed that there was no significant difference in potential publication bias on the results of our meta-analysis.

All the included studies scored 6 or more stars on the modified Newcastle–Ottawa scale, with no change in significance in the survival data by the sensitivity analysis. Egger linear regression method of 1-year DFS (*P* = 0.666; **Figure 4A**), 3-year DFS (*P* = 0.963; **Figure 4B**), 5-year DFS (*P* = 0.420; **Figure 4C**), 1-year OS (*P* = 0.579; **Figure 4D**), 3-year OS (*P* = 0.157; **Figure 4E**) and 5-year OS (*P* = 0.241; **Figure 4F**), revealed no significant difference in potential publication bias on the results of our meta-analysis.

## Discussion

This meta-analysis selected and summarized high-quality studies that compared the survival outcomes of rHCC and nrHCC patients. All the included studies had a case-matched design, and they were of high quality according to the PSM. The present meta-analysis revealed that the rupture was a poor factor of survival in HCC; the DFS and OS in patients with rHCC were shorter than those of nrHCC patients who underwent hepatic resection.

The information was limited when the rupture occurred in HCC patients, and diagnosis was frequently missed or delayed to allow the determination of the treatments to resolve emergent and complicated situations. Although most patients with spontaneous rupture of HCC have a higher tumor stage and worse liver function than those with nrHCC, previous studies demonstrated that emergency or staged hepatectomy should be performed in these patients (5, 25). A recent systematic review revealed that perioperative morbidity and in-hospital mortality rates were comparable between rHCC and nrHCC, indicating that partial hepatectomy was not associated with an increased perioperative risk for rHCC (10, 26), and radical resection may be the most important factor associated with long-term survival in patients with rHCC as suggested by the multivariate analysis (21, 22). Some studies reported that patients in the rHCC group have a worse OS, which may be due to the higher proportion of patients with worse liver function and higher tumor burden at baseline in the rHCC group compared with those in the nrHCC group (10, 11, 27). This result was also confirmed by another meta-analysis, in which the OS and DFS of rHCC patients are significantly shorter than those of nrHCC patients (13).

Recent studies reported that the length of OS is similar in patients with rHCC and those with nrHCC, especially among those with similar baseline tumor stage and liver function (6, 12), after adjusting for the incidence of these known prognostic factors. These reports suggest that the rupture itself may not be a prognostic factor for unfavorable OS (7), and rHCC should not be considered a “singular clinical entity” with a “rupture” event considered as the only determining prognostic factor (26). Thus, the pattern of tumor recurrence after partial hepatectomy is also controversial, and the biggest disagreement is about whether the rupture of HCC lead to a higher incidence of peritoneal dissemination or not. Previous studies reported that patients with peritoneal metastasis after hepatectomy had no previous experience of HCC rupture (28) and that the pattern of recurrence after hepatectomy is similar between rHCC and nrHCC (12, 22). In addition, peritoneal metastasis is not an independent prognostic factor (29). Therefore, the long-term cause of mortality is related to cancer recurrence and metastasis, and various therapeutic strategies have different effects on OS (26). Thus, the impact of rHCC on survival remains a debate among published studies.

The present study consisted of a meta-analysis of 6 studies (7, 20–24) that used PSM to reduce the bias between the rHCC and nrHCC group, which was an approach allowing to evaluate the true impact of rupture on survival. The factors associated with preoperative and postoperative prognosis such as tumor characteristics, Child-Pugh status, alpha-fetoprotein levels, and recurrence pattern were well-balanced among the included studies. Despite that, the DFS and OS remained worse in the rHCC group than in the nrHCC group, with no heterogeneity. Therefore, our study actually demonstrated that rupture was a risk factor for long-term prognosis in HCC patients after hepatectomy.

A recent meta-analysis (13) including 21 observational articles with 57,241 cases achieved a similar result. However, a high heterogeneity characterizes these studies because of the unbalanced baseline and pooling low-quality studies (30). The unbalanced and irrelevant baseline data like sample proportion, gender ratio, liver cirrhosis, liver function and tumor size and the high heterogeneity in the previous meta-analysis are evident, leading to unreliable statistical results; various subgroup analyses were performed to investigate whether the risk effect of rHCC varied among various subgroups, however, even the subgroup analysis did not solve the situation of high heterogeneity. The randomization of the treatment ensures that the treatment status is not confounded with complex baseline characteristics. Thus, the effect of the treatment on the outcomes is estimated by the direct comparison of the outcomes (31). It is difficult to perform a randomized method for the treatment because of the emergency situation and complex complications of the spontaneous rupture of HCC. The PSM method can achieve a “quasi-randomization” effect in non-randomized controlled studies that cannot be randomized during the study design stage. Therefore, our comprehensive meta-analysis selectively pooled high-quality studies to obtain a more systematic and strength power of the results assessing the impact of rupture on long-term survival in HCC patients.

It is not surprising that different therapeutic strategies have different effects on the OS of rHCC patients, when stratified by treatment modality, conservative approach and trans-arterial embolization (TAE)/trans-arterial chemoembolization (TACE) associated with the worst 1- and 3-year OS, whereas patients managed with emergency or staged hepatectomy have the most favorable outcomes (26, 32). Staged hepatectomy can reduce the intraoperative bleeding, intraoperative blood transfusion volume, and the 30-day mortality rate in spontaneous rHCC as well as increase the short- and long-term survival rates (32, 33). However, emergency hepatectomy has lower postoperative peritoneal dissemination rates than staged hepatectomy (32, 34, 35). Peritoneal dissemination is difficult to avoid, and a large amount of distilled water or hyperthermic intraperitoneal chemotherapy have been used for peritoneal irrigation (24). Recently, a large cohort study from France suggested that the surgical management of resectable peritoneal metastasis may provide a survival benefit for highly selected patients with exclusive peritoneal metastasis (36).

Another important factor of survival is the adjuvant treatment after curative hepatectomy. However, only limited data are available so far regarding the safety and efficacy of sorafenib in rHCC patients. Data from a single-institution showed that sorafenib use is feasible in patients with rHCC (37), since cumulative survival rates at 4, 8 and 12 months are higher in the surgery plus sorafenib group than in the surgery only group. Postoperative TACE can be used as adjuvant therapy to prevent the recurrence after hepatectomy (38), although perioperative TACE decreases intrahepatic metastasis but increases peritoneal dissemination in rHCC. Recently, Huang A et al. (32) found that adjuvant TACE confers a survival benefit in patients with high risk of recurrence (multiple tumors, as well as micro- and macro-vascular invasion). However, the above results should be interpreted with caution, since the sample of studies was limited (32, 36–38). Few studies have focused on the treatment of rHCC survivors, and subsequent treatment strategies are mainly determined clinically based on the tumor burden of rHCC survivors after the recurrence. Targeted therapies combined with immunotherapy have attracted a huge amount of interest in advanced HCC, leading to new strategies for the management of HCC patients (39). Taken together the real-life experience with systemic therapy in HCC patients confirms the safety of the treatment in patients with advanced HCC stages and even with reduced liver function.

The present meta-analysis has several limitations that should be mentioned. All the included studies had a retrospective design and are therefore subjected to potential confounders because of the lack of available randomized data, which likely caused selection bias. Second, patient outcomes could be influenced by variations among the studied populations, including disease stage, surgical techniques, and follow-up protocols, although studies that used the PSM methods were included. All studies included in our meta-analysis were from Asia, the most likely reason was the rHCC had variable incidence with reported rates of less than 3% in Western countries, and studies about rHCC were less common in Western centers (26). In the systematic search of meta-analysis, there was no comparative study between rHCC and nrHCC from the Western center, which likely caused selection bias. Sada et al., 2016 (21) and Tanaka et al., 2016 (7) investigated surgical procedures including one-stage and staged hepatic resection, Lee HS et al., 2014 (22) reported staged hepatic resection only, and Zhu Q et al., 2019 (20), Ruan S et al., 2020 (24) and Chua DW et al., 2019 (23) did not mention the surgical procedures used in their cohorts. Furthermore, the quality of the included studies was high according to the Newcastle–Ottawa scale, however, the size of the current study cohort was relatively small, thus reducing the quality of the conclusions.

Despite those limitations above, the present study is the first meta-analysis with balanced baseline that, to our knowledge, evaluated the influence of tumor rupture on the length of survival of HCC patient after hepatectomy. Indeed, it revealed that DSF and OS might be significantly shorter in patients with rHCC, thus highlighting the need for further well-designed clinical trials.

## Conclusion

The present meta-analysis indicated that spontaneous rupture of HCC is a predictor of poor survival, despite the need for higher-quality data. Surgical interventions provide a more favorable long-term outcomes, thus, radical resection should be the first choice when liver function is tolerated for respectable rHCC.

## Data Availability Statement

The raw data supporting the conclusions of this article will be made available by the authors, without undue reservation.

## Author Contributions

All authors contributed to the study concept, design, data interpretation, and discussion. XH, FL contributed to the screening and data collection. LX and ZH contributed to the assessment of the included article. XB and XY contributed to the data analysis. XH, XL contributed to the writing of the manuscript. XC and YN contributed to provide expert insight into the revision of the manuscript and being as corresponding author. CJ is responsible for the re-revision of the article. All authors approved the final version of the reports.

## Funding

This research supported by Sanming Project of Medicine in Shenzhen(No. SZSM202011010; No.SZSM201812079), Shenzhen Foundation of Science and Technology (No. JCYJ20170817172116272) and Shenzhen High-level Hospital Construction Fund.

## Conflict of Interest

The authors declare that the research was conducted in the absence of any commercial or financial relationships that could be construed as a potential conflict of interest.

## Publisher’s Note

All claims expressed in this article are solely those of the authors and do not necessarily represent those of their affiliated organizations, or those of the publisher, the editors and the reviewers. Any product that may be evaluated in this article, or claim that may be made by its manufacturer, is not guaranteed or endorsed by the publisher.

## Supplementary Material

Supplementary Figure 1 Forest plots for population characteristics of the included studies. Meta-analysis using the fixed effects model found no significant difference between rHCC and nrHCC groups in **(A)** sex ratio (OR, 1.27, 95%CI: 0.80–2.04, *P* = 0.31, *I* ^2^ = 0), **(B)** age (WMD, -0.62, 95%CI: -1.96–0.72, *P* = 0.37, *I* ^2^ = 0), **(C)** rate of liver cirrhosis (OR, 0.98, 95%CI: 0.64–1.50, *P* = 0.92, *I* ^2^ = 0) and **(D)** rate of Child-Pugh classification A (OR, 0.71, 95%CI: 0.39–1.31, *P* = 0.27, *I* ^2^= 0).

Supplementary Figure 2 Forest plots for population characteristics of the included studies. Meta-analysis using the fixed effects model found no significant difference between rHCC and nrHCC groups in **(A)** rate of macrovascular invasion (OR, 1.10, 95%CI: 0.73–1.66, *P* = 0.65, *I* ^2^ = 0), **(B)** rate of microvascular invasion (OR, 1.22, 95%CI: 0.84–1.79, *P* = 0.30, *I* ^2^ = 0), **(C)** rate of solitary nodule (OR, -0.04, 95%CI: -0.12–0.04, *P* = 0.31, *I* ^2^ = 30%) and **(D)** tumor size (WMD, 0.22, 95%CI: -0.43–0.86, *P* = 0.51, *I* ^2^ = 4%).
