# Supplementary figures and images for: Survival of Patients Subjected to Hepatectomy After Spontaneous Rupture of Hepatocellular Carcinoma: A Meta-analysis of High-quality Propensity Score Matching Studies
Source: Front Oncol. 2022 May 19;12:877091. doi: 10.3389/fonc.2022.877091 (PMC9160741; doi:10.3389/fonc.2022.877091)

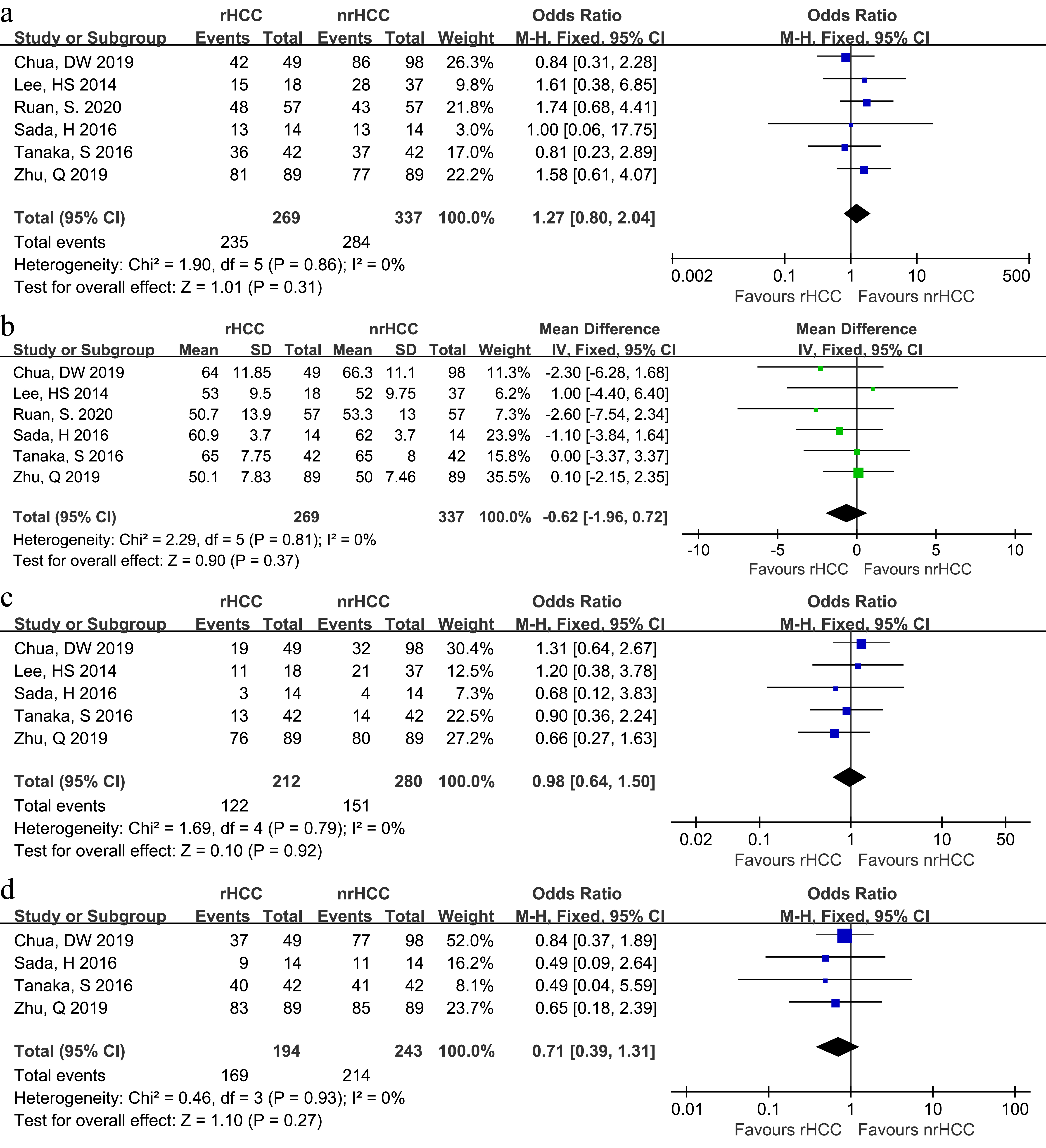

Supplement: Supplementary Figure 1 — Forest plots for population characteristics of the included studies. Meta-analysis using the fixed effects model found no significant difference between rHCC and nrHCC groups in (A) sex ratio (OR, 1.27, 95%CI: 0.80–2.04, P = 0.31, I 2 = 0), (B) age (WMD, -0.62, 95%CI: -1.96–0.72, P = 0.37, I 2 = 0), (C) rate of liver cirrhosis (OR, 0.98, 95%CI: 0.64–1.50, P = 0.92, I 2 = 0) and (D) rate of Child-Pugh classification A (OR, 0.71, 95%CI: 0.39–1.31, P = 0.27, I 2= 0). [file Image_1.jpeg]

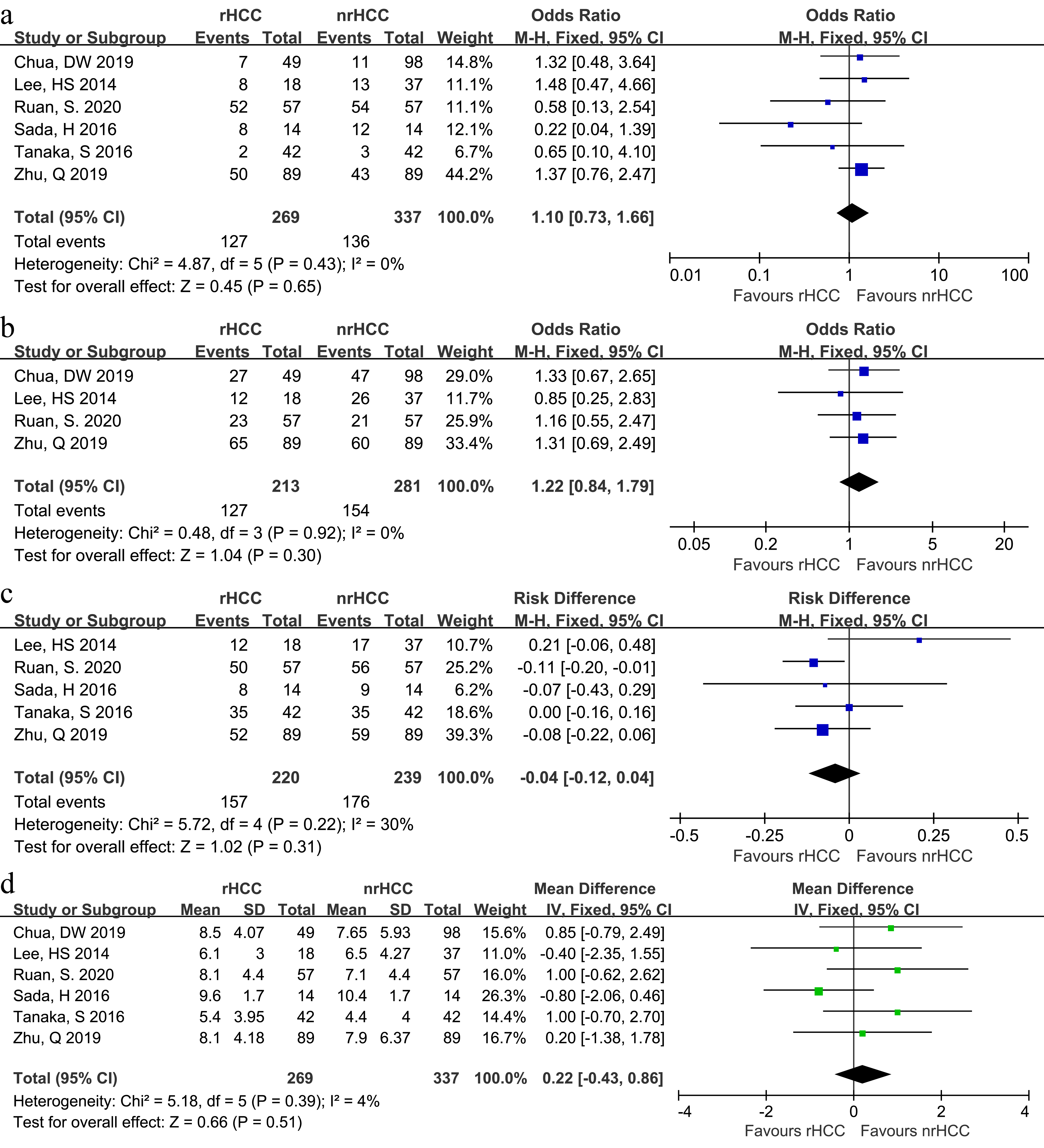

Supplement: Supplementary Figure 2 — Forest plots for population characteristics of the included studies. Meta-analysis using the fixed effects model found no significant difference between rHCC and nrHCC groups in (A) rate of macrovascular invasion (OR, 1.10, 95%CI: 0.73–1.66, P = 0.65, I 2 = 0), (B) rate of microvascular invasion (OR, 1.22, 95%CI: 0.84–1.79, P = 0.30, I 2 = 0), (C) rate of solitary nodule (OR, -0.04, 95%CI: -0.12–0.04, P = 0.31, I 2 = 30%) and (D) tumor size (WMD, 0.22, 95%CI: -0.43–0.86, P = 0.51, I 2 = 4%). [file Image_2.jpeg]
